# Reaping the Whirlwind

**Published:** 1917-07

**Authors:** 


### "REAPING THE WHIRLWIND"

The inevitable has happened. When the first flush of enthusiasm over the value of the X-ray in root filling was at its height, and following this when the first terror-stricken stampede for cover took place under the lurid announcement of the terrible evils which lurked in the wake of all the root filling of the past; there were certain men in the profession who said some very foolish things, and some of these foolish things got into print. The result is precisely what might have been expected. When men got up before societies and waved their hands excitedly and declared that dentists in the future would lay themselves liable for damage suits unless they filled root canals according to the orthodox methods laid down by the speakers, they were not only saying some foolish things, but they were saying some dangerous things. They were unwittingly laying pitfalls for their fellow practitioners and possibly for themselves.

Everyone has heard of the process, "Sowing the wind and reaping the whirlwind." These men were sowing the wind—*much of it was wind*—and now comes the whirlwind. What better bait could an irresponsible lawyer want than a statement like that to start all sorts of unjust damage suits against dentists? And that is precisely what has been done. We are informed that no fewer than thirty-two damage suits have been started in New York alone. Who is to be blamed? Mostly the men who foolishly proclaimed the possibility of such a thing in the first place. It was foolish from every point of view—foolish because of the invitation it gave to trouble, and more foolish because of the fact that the statements made as to the evil effects of root canal work had not by any means been established, and we are going to be heretical enough to say that they are not definitely established yet. Who shall say which technic is safe and which not? And who with a sweep of the hand shall attempt to bring discredit on the conscientious efforts of the men who for years have made an honest attempt to save pulpless teeth for their patients? Are dentists to be dubbed as criminals because they can not all see things in the same light, and forsooth because they sometimes fail in their operations? Who has ever claimed infallibility for dentists?

And in what other calling are men taken to task because they are not infallible? Mistakes are made by men in every pursuit because the men following those pursuits are human, and if criminal proceedings were instituted against all men who make mistakes, the courts would be full to overflowing.

No conscientious man wishes to condone willful carelessness in the management of pulpless teeth; no man denies the sacred obligation under which a practitioner is placed to his patient when he is rendering service, and all men will agree that the patient is entitled to the greatest care and a reasonable degree of skill. But to sue a dentist for damages in a case of pulp canal filling on the evidence presented by X-ray pictures is merely a species of blackmail, and is calculated to work a serious injustice on the practitioner.

We ventured a prediction when the indiscreet utterances previously referred to were made that they would open up possibilities of persecution of dentists, and now that this prediction has been fulfilled, we will venture another. Just so surely as these damage suits become general, just so surely will dentists be driven to abandon the effort to save pulpless teeth, and will condemn them to the forceps. We are aware that there are some men in the profession who will rise up and hail this as a fortunate circumstance. We are not of that number.—Editorial by C. N. Johnson in *Dental Review*.

